# Decision tree model to assess consequences and costs associated with therapy administration pathways for patients with HER2+ breast cancer in Italian oncological centers

**DOI:** 10.1371/journal.pone.0351548

**Published:** 2026-07-24

**Authors:** Giorgia Gribaudo, Carla Fornari, Ippazio Cosimo Antonazzo, Daniele Generali, Carlo Carnaghi, Alessandro Galimberti, Patrizia Nardulli, Monica Calamai, Davide Marchesini, Lorenzo Giovanni Mantovani, Paolo Angelo Cortesi

**Affiliations:** 1 Research Center on Public Health Centro Studi Dipartimentale in Sanità Pubblica (CESP), University of Milan-Bicocca, Monza, Italy; 2 University of Bologna, Bologna, Italy; 3 Department of Environmental and Prevention Sciences, University of Ferrara, Ferrara, Italy; 4 Department of Medicine, Surgery and Health Sciences, University of Trieste, Trieste, Italy; 5 Breast Multidisciplinary Unit, ASST of Cremona, Cremona, Italy; 6 UO Oncologia e Ematologia, Humanitas Istituto Clinico Catanese, Misterbianco, Catania, Italy; 7 Project Manager, IRCCS Humanitas Research Hospital, Rozzano, Italy; 8 SC Pharmacy, IRCCS Istituto Tumori “Giovanni Paolo II”, Bari, Italy; 9 Direttore generale Ausl Ferrara, Commissario Straordinario Azienda Ospedaliera Universitaria Ferrara, Ferrara, Italy; 10 Fondazione Charta, Milano, Italy; 11 Public Health Laboratory, IRCCS Auxologico, Milan, Italy; University of Salerno, ITALY

## Abstract

**Background and aims:**

Human Epidermal Growth Factor Receptor 2-positive (HER2+) breast cancer poses significant therapeutic challenges, particularly concerning treatment administration pathways and their associated costs. This study evaluates the managerial and economic impacts of different therapeutic administration scenarios for HER2-positive breast cancer patients, focusing on optimizing hospital workflows, resource utilization, and patient outcomes in Italian oncological centers.

**Methods:**

A decision tree model was developed to simulate and compare five treatment administration pathways: Standard, Drug-Change, Drug Day, Dedicated Ambulatory, and Optimal Pathway scenarios. The model integrates patient and healthcare professional (HCP) activity and waiting times, infusion chair occupation, and direct and indirect costs. Sensitivity analyses assessed variability in model outcomes.

**Results:**

Switching from endovenous (EV) to subcutaneous (SC) administration substantially reduced patient throughput times and HCP workloads. The Optimal Pathway scenario yielded the highest resource optimisation, reducing HCP activity time by up to 48 hours, infusional chair occupational time by up to 150 hours, and patients’ total time by up to 753 hours per 100 patients monthly. Cost analyses indicated significant savings in both direct and indirect cost for all the proposed scenarios in comparison to the Standard one.

**Conclusion:**

The adoption of SC formulations and innovative pathway optimizations enhances treatment organizational efficiency and reduces both direct and indirect costs. These findings underscore the value of tailored approaches to administration based on the structural and organizational characteristics of individual oncology centers, aligning with current Italian healthcare reforms.

## Introduction

Breast carcinoma results from the uncontrolled proliferation of epithelial cells in the mammary gland, which transform into malignant cells and gain the ability to detach from their original tissue, invade adjacent tissues, and, over time, spread to distant organs. Excluding non-melanoma skin cancers, breast cancer is the most frequently diagnosed malignancy among women in Italy, representing 40% of cancers in the 0–49 age group, 35% in the 50–69 age group, and 22% in women over 70 [1]. Breast cancer accounts for 30.3% of all female cancers, with an estimated 55,900 new cases diagnosed in 2023, marking an increase from 2022. It remains the most prevalent neoplastic disease among women, with 834,200 cases, constituting 43% of the total cancer prevalence. Mortality in 2022 was estimated at 15,500 deaths [2].

Human epidermal growth factor receptor 2 (HER2) tyrosine kinase is overexpressed in 20% of breast cancers and associated with a less favorable prognosis than HER2-negative disease. This receptor is a target for systemic therapies, which include anti-HER2 monoclonal antibodies, such as trastuzumab and pertuzumab. These are standard treatments for HER2-positive breast cancer in the neoadjuvant, adjuvant, and first-line metastatic settings [3–5] and they are administered subcutaneously or intravenously.

These treatments are typically administered in a day hospital (DH) or outpatient setting, allowing patients to receive care and return home the same day. This approach is common in both the neo/adjuvant and metastatic settings, where patients receive therapy and are discharged on the same day.

Given the increase in early diagnosis achieved through mammography screening and advancements in treatment options for patients with HER2+ breast cancer, the Italian NHS (SSN) needs to identify the best pathways for the management of these patients.

Recent advancements in anti-HER2 therapies have not only improved patient outcomes but have also raised significant questions regarding associated costs. As treatments for the metastatic disease increasingly evolve into chronic management and curative potential emerges in the adjuvant setting, balancing therapeutic efficacy, economic sustainability, and resource efficiency remains a critical challenge [6].

The centers involved in receiving and treating these patients on the Italian territory are very heterogeneous: monoclonal antibody therapy is administered both in hospital and, potentially, in territorial outpatient settings. It is therefore of considerable importance to have a tool that allows individual centers to simulate which is the most suitable operational model based on their structural characteristics.

The main aim of this project was to develop a decision-support tool comparing different treatment administration pathways for patients with HER2+ breast cancer in Italian oncological centers. The tool focuses on the administration of trastuzumab+pertuzumab therapy in a hospital setting and evaluates the organizational and economic implications of alternative administration scenarios. Specifically, it compares the current treatment administration pathway to alternative scenarios in which the subcutaneous administration is introduced, together with pathway optimization strategies. The analysis was designed to assess changes in resource utilization, healthcare professional (HCP) activity time, patient and caregiver time.

## Methods

### Administration pathway description

The day-hospital administration pathway involves consecutive activities spread by variable waiting times [7]. The sequence may vary depending on the organizational strategy of the center, but the described pathway assumes that all the activities for therapy administration are conducted over a single day. The pathway is divided into six phases, each related to a specific activity performed in the day-hospital treatment pathway:

1. the first phase is “Acceptance”, which includes welcoming, registering, and directing the patient within the center;
2. the second phase, “Blood sample and reporting”, involves the performance of pre-treatment blood tests (e.g., liver and kidney function, blood count, electrolytes) and reporting;
3. the third phase, “Visit”, refers to the pre-administration visit, which is necessary to verify patients’ health status before therapy is administered;
4. the fourth phase, “Drug Preparation”, involves drug preparation according to priorities, timing, and preparation criteria established by each center;
5. the following phase is the “Drug administration”, in which patients are seated on infusion chairs and are assisted during the treatment administration;
6. in the last phase, “Observation and Discharge”, patients are carefully observed and informed about self-monitoring activities and the reporting of any adverse event.

The day-hospital administration pathway is shown in S1 Fig.

### Model description

**Fig 1:**
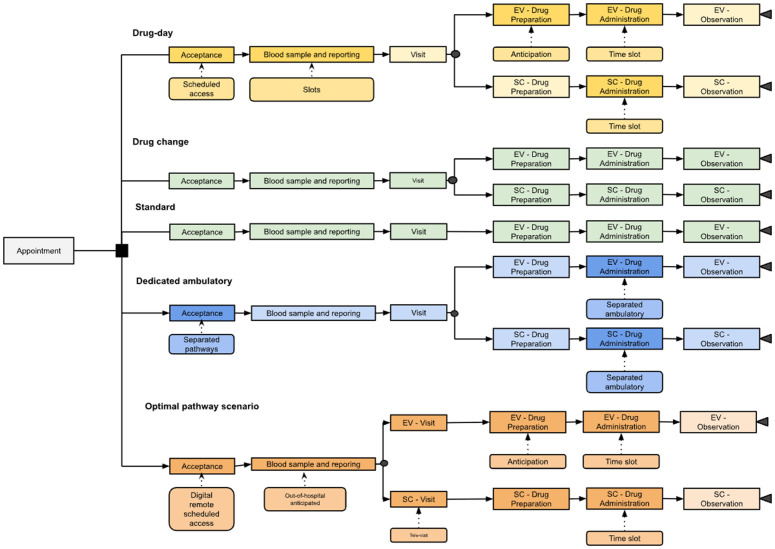
Decision tree model showing the different treatment administration pathways for patients with HER2+ breast cancer in Italian oncological centers.

To simulate different treatment administration pathways for patients with HER2+ breast cancer in Italian oncological centers, we adopted a decision tree model in Microsoft @Excel. The decision tree compares the actual treatment administration pathway (Standard Scenario) to four alternative scenarios, including improvement actions to optimize the time and effort required by centers, HCPs, patients, and caregivers, as shown in Fig 1.

- The Standard scenario presents the previously mentioned steps following each other: acceptance, blood sample and reporting, and visit. These are followed by drug preparation and administration of the endovenous (EV) formulation of pertuzumab and trastuzumab. The last phase is observation and discharge, which is also influenced by the drug EV formulation.
- The Drug-change scenario introduces the subcutaneous (SC) formulation of the pertuzumab+trastuzumab fixed dose combination (PH FDC SC, Phesgo®). The first 3 steps do not include any improvement action, then the decision tree presents a chance node that can lead to SC or EV drug preparation and administration, followed by the last phase, the observation and discharge one.
- The Drug day scenario presents four improvement actions in addition to SC use. The acceptance phase is improved by the application of the “scheduled access”: the patient access time is scheduled in detail and the patient is educated to arrive at a previously defined access time. Patient access scheduling should also consider the therapy and drug formulation that patients will receive, to guarantee their most efficient alternation, leading to a minor patient waiting time and minor activity times for both patients and HCPs. In the blood sample and reporting step, “time slots” improvement action is implemented: laboratories are set to receive specific analysis sets for patients in treatment, therefore dedicated time slots for blood samples and their reporting are planned to waste as little time as possible in the laboratory. Then, the scenario has the same chance node that divides the pathway into two arms: patients receiving the EV formulation and those receiving the SC one. In the drug preparation phase of the EV arm, the “anticipation” improvement action is applied, and the hospital pharmacy can prepare the pertuzumab and trastuzumab infusion bags in advance. Regarding the drug administration phase, both EV and SC arms implement “time slots” improvement action, which consists of a drug administration schedule that takes into account the number of patients who will receive the EV and the SC formulation and alternates them in the infusion chair to maximize their occupancy and reduce waiting times to minimum.
- The Dedicated ambulatory scenario includes improvement actions to optimize the available spaces in relation to dedicated use for specific types of patients and treatments. It starts with the improvement action “separated pathways” in the access phase, which presume to have the possibility of splitting the patients’ flows based on the drug formulation that they will receive. This pathway is also divided by the chance node between EV and SC formulations at the drug preparation step. In both arms, the drug administration phase is associated with the “separated ambulatory” implementation action, which allows splitting EV and SC patients into different spaces and programming the infusion chair occupational time depending on the drug formulation timing.
- The Optimal pathway scenario implements all the possible improvement actions as much as possible. It starts with an optimized acceptance phase through the digital remote scheduled access, in this way, not only the patients arriving are scheduled, but patients are also able to access the acceptance process through digital remote tools, allowing them to save time once they arrive at the center. The blood sample and reporting phase, when possible, is anticipated on a previous day and it is performed in a different setting, to save time on the therapy day and to allow patients to access the blood sample procedure in a closer location. In the visit phase, this scenario presents the chance for patients in SC formulation to benefit from the “tele-visit” implementation action. Moreover, the EV arm benefits from the “anticipation” action associated with the drug preparation phase, while both SC and EV arms benefit from the “time slots” improvement action, similar to what happens in the drug day scenario.

In all the scenarios presented in the model, no improvement actions have been defined and implemented for the observation and discharge phase, given that this phase timing is strictly connected to the nature of the drug administered. As previously mentioned, only the switch from an EV formulation to an SC one can reduce the necessary observation time. For all the scenarios, it is recommended to look at the implementation actions as an improvement of the synergistic system of the pathway.

The analysis was conducted from both the Italian National Health Service (NHS), the oncology center (provider), and the societal perspective, in order to capture the different components of resource utilization and costs associated with the administration pathways.

The model simulates both a single treatment access (daily perspective) and a monthly treatment scenario, based on a standard 21-day therapeutic cycle, corresponding to the usual administration schedule for trastuzumab and pertuzumab therapies..

### Model outcomes and costs

The analysis reports costs and outcomes separately, with the aim of supporting decision-making on evaluating organizational and economic implications of alternative treatment administration pathways; rather than comparing clinical effectiveness of therapies. This approach is based on the model’s assumption of equal clinical effectiveness between IV and SC therapies, shifting the focus away from a direct comparison of clinical efficacy. The model estimates organisational efficiency outcomes of the treatment administration pathway for all the simulated scenarios: the **patient crossing time**, both considered as a whole (*total*) and divided into *activity* and *waiting* time; the **HCP activity time**, both the overall one (for all the professional figures involved in the treatment pathway) and separately for each figure, administrative personnel, nurse, pharmacist, clinician, and the **occupation time of infusion chairs**. Moreover, the model computes the **patient total times** by adding the travel time to reach the place of treatment administration to the crossing time, and the caregiver time taken to assist the patient during the day. All times are reported both in minutes and hours.

Other outcomes analyzed are:

- the percentage of patients with **PICC** or **PORT catheter;**
- the number of **adverse events** (when the whole month is simulated): pocket infections with fever or catheter-related bloodstream infections (CRBI) and catheter-related upper extremity vein thrombosis (UEDVT).

Costs are reported in euros (€) and divided into direct healthcare and indirect costs:

- **direct healthcare costs** include HCP work in the treatment pathway (as a whole and by each professional figure), infusion c.hair occupation, pharmacological treatment purchase, and adverse events management.
- **indirect costs** are related to productivity losses for patients and caregivers.

All outcomes and costs are reported as values and differences to the standard reference scenario.

### Model inputs

The oncological center dimension – small, medium, big – defines the number of patients that daily access the simulated oncological center. The model simulates outcomes and costs for 1 daily treatment access or for a therapeutic cycle (N daily access every 21 days). In this simulation, we simulate treatment pathways considering both 1 and 100 patients for 1 daily access and during a month of a therapeutic cycle with 21-days infusion interval. Model inputs were derived from a combination of published literature, real-world data [7–10], and structured expert opinion, particularly for parameters related to workflow organization and healthcare professional activity time, which are not routinely available in administrative datasets.

All the population input parameters are reported in Table 1.

**Table 1:** Population and main general parameters.

Parameter Description	Value	Source
Percentage of patients in EV therapy	0.292	PHASTER Project 2023 [8]
Percentage of patients needing blood sample exams	0.150	Expert opinion
Patients mean weight (Kg)	71.5	Zambelli et al.2023 [9]
Catheter Use		
PORT – EV administration	0.675	PHASTER Project 2023 [8]
PORT – SC administration	0.070	PHASTER Project 2023 [8]
PICC – EV administration	0.127	PHASTER Project 2023 [8]
PICC – SC administration	0.000	PHASTER Project 2023 [8]
Risk of Adverse Events		
Pts with PICC -Rate of Pocket Infections or CRBI (x 1 catheter day)	0.000063	Bertoglio et al. 2020 [10]
Pts with PICC – Rate of Thrombotic events (x 1 catheter day)	0.000105	Bertoglio et al. 2020 [10]
Pts with PORT -Rate of Pocket Infections or CRBI (x 1 catheter day)	0.000063	Bertoglio et al. 2020 [10]
Pts with PORT – Rate of Thrombotic events (x 1 catheter day)	0.000105	Bertoglio et al. 2020 [10]
Percentage of worker patients	0.44	PHASTER Project 2023 [8]
Percentage of patients with caregiver	0.65	PHASTER Project 2023 [8]
Percentage of worker caregivers	0.65	PHASTER Project 2023 [8]

We populated crossing times for each phase of the treatment pathway in the Standard and Drug-Change scenarios by using the data reported in the expert opinion research [7] and in the Phaster Project [8], reported in Table 2.

**Table 2:** Crossing times in the standard and drug-change scenarios.

*Treatment Pathway Phases*	*Parameter value (minutes)*				*Sources*
	*Patient Active Time*	*Patient Waiting Time*	*HCP Active Time*	*Infusion Chair Occupation Time*	
Acceptance	8.72	50.40	7.00	–	PHASTER Project 2023 [8]
Blood Sample and Reporting	10.64	26.82	12.00	–	PHASTER Project 2023 [8]
Visit	17.88	65.20	18.00	–	PHASTER Project 2023 [8]
*EV drug administration*					
Drug Preparation	–	17.50	7.00	–	Deloitte Consulting 2023 [7] and PHASTER Project 2023 [8]
Drug Administration	96.98	–	25.00	102.48	Deloitte Consulting 2023 [7] and PHASTER Project 2023 [8]
Observation	110.00	–	–	–	Deloitte Consulting 2023 [7] and PHASTER Project 2023 [8]
*SC drug administration*					
Drug Preparation	–	5.00	2.00	–	Deloitte Consulting 2023 [7] and PHASTER Project 2023 [8]
Drug Administration	7.50	–	13.00	13.28	Deloitte Consulting 2023 [7] and PHASTER Project 2023 [8]
Observation	34.50	–	–	–	Deloitte Consulting 2023 [7] and PHASTER Project 2023 [8]

Parameters related to the effect of previously described improvement actions on crossing times are defined by expert opinions and are reported in Table 3.

**Table 3:** Parameter of crossing times reduction related to implemented improvement actions.

*Scenario*	*Improvement Action*	*Administration Type*	*% of reduction*			*Source*
			*Patient Active Time*	*Patient Waiting Time*	*HCP Active Time*	
*ACCEPTANCE Phase*						
Drug-day	Scheduled access	EV/SC	7.5%	17.3%	5.0%	Expert opinion
Dedicated Ambulatory	Separated pathways	EV/SC	8.8%	7.8%	0.0%	Expert opinion
Optimal pathway	Digital remote scheduled access	EV/SC	60.5%	67.5%	32.5%	Expert opinion
*BLOOD SAMPLE and REPORTING Phase*						
Drug-day	Time slots	EV/SC	3.3%	6.3%	1.3%	Expert opinion
Dedicated Ambulatory	None	EV/SC	0%	0%	0%	Model assumption
Optimal pathway	Anticipation	EV/SC	100%	100%	100%	Model assumption
*VISIT Phase*						
Drug-day	None	EV/SC	0%	0%	0%	Model assumption
Dedicated Ambulatory	None	EV/SC	0%	0%	0%	Model assumption
Optimal pathway	None	EV	0%	0%	0%	Model assumption
	Tele-visit	SC	43.0%	77.5%	22.2%	Expert opinion
*DRUG PREPARATION Phase*						
Drug-day	Anticipation	EV	–	72.5%	5.0%	Expert opinion
	None	SC	0%	0%	0%	Model assumption
Dedicated Ambulatory	None	EV	0%	0%	0%	Model assumption
	None	SC	0%	0%	0%	Model assumption
Optimal pathway	Anticipation	EV	–	72.5%	5.0%	Expert opinion
	None	SC	0%	0%	0%	Model assumption
*DRUG ADMINISTRATION Phase*						
Drug-day	Time slots	EV	10.0%	–	10.0%	Expert opinion
	Time slots	SC	29.0%	–	30.5%	Expert opinion
Dedicated Ambulatory	Separated Ambulatory	EV	12.5%	–	7.5%	Expert opinion
	Separated Ambulatory	SC	20.5%	–	28.0%	Expert opinion
Optimal pathway	Time slots	EV	10.0%	–	10.0%	Model assumption
	Time slots	SC	29.0%	–	30.5%	Model assumption

Observation Phase is not included in the table because no improvement actions are applied.

In detail, “scheduled access” implementation in the acceptance phase reduces HCP active times by 5.0% patient’s active time by 7.5% and patient waiting time by 17.3% (ref. times for standard scenario) in the drug-day scenario. In the dedicated ambulatory scenario, the “separated pathways” improvement action reduces patient waiting and active time by 8.8% and 7.8% respectively. The activation of a digital remote scheduled access reduces HCP active time by 32.5% and patient waiting and active time by 67.5% and 60%, respectively.

In the blood sample and reporting phase scheduled time slots slightly reduce all crossing times in the drug-day scenario. In the optimal pathway scenario, anticipation of blood sample can be performed for 52% of patients; for these patients, no crossing times were included (100% reduction), and for the others crossing times are equal to those in the standard pathway.

Tele-visit improvement action is simulated only in the optimal pathway scenario for 41% of patients with SC administration (expert opinion) and reduces their active time by 43% and the waiting time by 77.5%. Moreover, the model assumes that these patients will probably receive therapy administration in a center near their home, so it assumes their travel time is reduced by 70%.

In the drug preparation phase of the drug-day and optimal pathway scenarios, anticipation of EV drug formulation is implemented and is supposed to reduce patient waiting time by 72.5%. This implementation action is applied only for the EV formulation because it is the one that requires bag preparation, whereas the SC formulation comes ready to administer, so there is no need or opportunity to anticipate preparation to earlier times.

Time slots and separated ambulatory actions reduce activity times by 10% and 12.5%, respectively, for patients in EV formulation and by 29% and 20.5%, respectively, for patients with SC formulation, when applied.

No improvement action is designed for the observation and discharge phase.

As direct healthcare costs, we considered the working cost for all the HCPs involved in the treatment pathway [11,12], the cost of infusion chair occupation [11], ex-factory treatment costs with disclosed and undisclosed discounts [13] and costs for adverse events management [14]. Indirect input costs considered were the working costs for patients or caregivers (work loss) [15,16]. All input cost parameters are illustrated in Table 4.

**Table 4:** Direct healthcare costs and indirect costs.

Parameter description	Unit measure	Value	Source
HCP work cost			
Clinician	€ per work hour (€/h)	47.48	Bergamaschi R. AboutOpen 2024; 11: 48–56 [11]
Nurse	€ per work hour (€/h)	24.20	Bergamaschi R. AboutOpen 2024; 11: 48–56 [11]
Pharmacist	€ per work hour (€/h)	42.30	Bergamaschi R. AboutOpen 2024; 11: 48–56 [11]
Administrative	€ per work hour (€/h)	21.00	Lazzaro et al 2023 [12]
Infusion Chair	(€/min)	0.07	Bergamaschi R. AboutOpen 2024; 11: 48–56 [11]
EV (Trastuzumab 600 mg)	€/mg	0.35	National mean (10/12/2024)
EV (Pertuzumab 420 mg)	€ per administration	1813.59	GU262, 09/11/2023 Ex-factory with discounts [13]
SC (T 600 mg + P 600 mg)	€ per administration	1911.20	GU168, 22/07/2025 Ex-factory with discounts [13]
Cost AE for CRBI	€ per event	5575.00	Mandolfo et al. 2019 [14]
Cost AE for Thrombotic event	€ per event	5575.00	Mandolfo et al. 2019 [14]
Working cost hour	€ per work hour (€/h)	14.71	Filippi et al. 2023 [15]. ISTAT 2013 actualized 2021 [16]

### Sensitivity analysis

A deterministic one-way sensitivity analysis (OWSA) was performed to assess the impact of varying one model parameter at a time on predicted costs and outcomes, while keeping all other parameters constant. The range of variation of each parameter was defined based on its distribution, using mean and standard error values and corresponding confidence intervals. Results were ranked according to their absolute impact and presented using a tornado diagram, highlighting the most influential parameters.

The outcomes included in the OWSA analysis were the patient total time and the HCP active time, while the costs included were the total direct cost and the total cost.

In addition, a probabilistic sensitivity analysis (PSA) was conducted to account for parameter uncertainty and to estimate the confidence intervals of the model outcome and cost estimates. After assigning a distribution and uncertainty range to each input parameter, a Monte Carlo simulation was conducted with repeated sampling sets of all inputs over 1000 simulation runs. Mean values and the 2.5 and 97.5 percentiles are reported. Notably, while the PSA was used to assess robustness, cost-effectiveness acceptability curves were not generated; this is because the analysis does not aim to estimate a summary cost-effectiveness metric, but focuses instead on evaluating separately the organizational and economic implications of alternative treatment administration pathways.

### Ethic statements

This article is based on mathematical modelling with inputs informed by previously conducted studies and does not contain any new studies with human participants or animals performed by any of the authors.

## Results

### Crossing times

The results of monthly crossing times for a sample of 100 patients with the probabilistic analysis are summarized in Table 5. Results per patient can be consulted in S1 Table.

**Table 5:** Monthly crossing times (hours) for 100 oncological patients in different pathways and in comparison with the current standard scenario.

Outcomes	Standard	Drug change (Δ Standard)	Drug Day (Δ Standard)	Dedicated ambulatory (Δ Standard)	Optimal pathway (Δ Standard)
Healthcare Professional Active Time, hours (IC), **Δ Standard**					
*Administrative* **Δ Standard**	16.68 (13.45; 20.11) Ref	16.68 (13.45; 20.11) **0.00** **(0.00; 0.00)**	15.85 (12.78; 19.11) **−0.83** **(−1.01; 0.67)**	16.68 (13.45; 20.11) **0.00** **(0.00; 0.00)**	11.26 (9.08; 13.58) **−5.42** **(−6.54; −4.37)**
*Nurse* **Δ Standard**	63.93 (53.10; 76.55) Ref	43.79 (38.13; 50.28) **−20.15** **(−30.54; −10.89)**	35.26 (30.59; 40.82) **−28.67** **(−38.75; −19.73)**	36.30 (31.49; 42.02) **−27.63** **(−37.62; −18.87)**	35.53 (28.95; 39.05) **−30.40** **(−40.44; −21.58)**
*Pharmacist* **Δ Standard**	16.65 (13.58; 19.95) Ref	8.25 (6.92; 9.61) **−8.39** **(−10.88; −5.99)**	8.01 (6.74; 9.31) **−8.64** **(−11.16; −6.23)**	8.25 (6.92; 9.61) **−8.39** **(−10.88; −5.99)**	8.01 (6.74; 9.31) **−8.64** **(−11.16; −6.23)**
*Clinician* **Δ Standard**	42.50 (34.40; 51.00) Ref	42.50 (34.40; 51.00) **0.00** **(0.00; 0.00)**	42.50 (34.40; 51.00) **0.00** **(0.00; 0.00)**	42.50 (34.40; 51.00) **0.00** **(0.00; 0.00)**	39.37 (31.80; 47.38) **−3.13** **(−4.11; −2.35)**
All **Δ Standard**	139.77 (124.54; 155.18) Ref	111.23 (100.18; 122.60) **−28.54** **(−38.51; −19.11)**	101.62 (91.27; 112.59) **−38.14** **(−48.12; −29.01)**	103.74 (93.21; 114.88) **−36.03** **(−45.96; −26.99)**	92.17 (82.60; 102.75) **−47.59** **(−58.02; −38.40)**
Infusion Chair Occupation Time, hours (IC), **Δ Standard**	243.64 (197.59; 291.79) Ref	93.64 (75.72; 112.06) **−150.01** **(−188.30; −115.91)**	93.64 (75.72; 112.06) **−150.01** **(−188.30; −115.91)**	93.64 (75.72; 112.06) **−150.01** **(−188.30; −115.91)**	93.64 (75.72; 112.06) **−150.01** **(−188.30; −115.91)**
Patient in-hospital Time, hours (IC), **Δ Standard**					
*Active Time* **Δ Standard**	560.99 (495.15; 633.77) Ref	282.11 (251.71; 316.78) **−278.88** **(−335.82; −224.88)**	193.32 (173.52; 213.95) **−367.67** **(−433.78; −308.04)**	192.58 (173.05; 213.08) **−368.41** **(−434.66; −308.78)**	174.72 (155.47; 195.18) **−386.27** **(−451.75; −326.03)**
*Waiting Time* **Δ Standard**	325.67 (288.25; 366.87) Ref	304.56 (266.83; 344.84) **−21.11** **(−28.18; −14.71)**	274.47 (239.24; 312.18) **−51.20** **(−60.05; −42.59)**	295.28 (258.48; 334.71) **−30.39** **(−37.23; −24.02)**	170.74 (145.69; 198.00) **−154.93** **(−176.61; −135.76)**
*Total Time* **Δ Standard**	886.66 (807.84; 967.69) Ref	586.67 (534.44; 638.19) **−299.99** **(−358.19; −244.88)**	467.80 (427.52; 508.62) **−418.86** **(−484.13; −356.72)**	487.86 (445.85; 530.59) **−398.80** **(−464.13; −338.19)**	345.46 (312.84; 380.33) **−541.20** **(−612.05; −473.75)**
Patient Total Time^1^, hours **Δ Standard**	1.123.80 (1.032.72; 1.214.49) Ref	823.81 (755.27; 895.61) **−299.99** **(−358.19; −244.88)**	704.94 (641.27; 768.96) **−418.86** **(−484.13; −356.72)**	725.00 (661.29; 790.31) **−398.80** **(−464.13; −338.19)**	370.99 (336.93; 406.52) **−752.81** **(−836.37; −679.12)**
Caregiver Total Time^1^, hours **Δ Standard**	734.25 (575.18; 887.69) Ref	538.26 (417.60; 644.79) **−195.99** **(−251.18; −145.36)**	460.60 (357.94; 550.30) **−273.65** **(−343.12; −209.23)**	473.71 (366.92; 566.98) **−260.54** **(−328.76; −197.93)**	242.35 (189.86; 292.04) **−491.90** **(−598.72; −381.73)**

Times are reported in hours.

^1^Including travel time.

The table shows how each scenario and its improvement actions impact crossing times, either those affecting the HCP or those involving patients and caregivers. The introduction of the SC formulation (Drug change vs Standard scenario) reduced most crossing times outcomes. HCP activity time is reduced by 29 hours for 100 patients treated (20%-time reductions); the figure most affected by SC formulation introduction is the nursing staff, saving 0.14 full time equivalent (FTE) staff (working 36 hours/month) per 100 patients/month with 1 therapy access since the drug formulation change affects drug administration phase, which is managed by nurses. Pharmacist’s crossing time is also affected in the drug preparation phase in which drug preparation times are reduced for SC formulation, saving 0.06 FTE staff. Moreover, infusion chair occupancy time decreases by 150 hours per 100 patients treated (62% reduction) by switching from EV to SC formulation. The infusion chair free time gained may be converted into 230 new therapy infusions assuming model input data for chair occupation time and drug formulation use distribution [8,9]. SC formulation introduction also affects patients’ crossing times reducing both activity and waiting times of 279 (50% reduction) and 21 (6%) hours, respectively. Patients’ and caregivers’ total times are consequently reduced by 26–27%.

All other introduced improvement actions in drug day, dedicated ambulatory and optimal pathways further reduce HCP activity times, but have a major impact on patients’ and caregiver crossing and total times. The results show that the most beneficial interventions in reducing crossing and total times are, in ascending order, those related to the scenarios: Drug change, Dedicated ambulatory, Drug Day, and Optimal pathway. The Optimal pathway scenario, is the one associated with the shortest total patient crossing time, 371 hours per 100 patients treated, “optimized acceptance phase” and “time slots” improvement actions reduced HCPs’ and patients’ crossing times, moreover the “external blood sample” improvement action when applicable erases all waiting and activity times of patients and HCPs associated with this phase since the perspective adopted by the analysis is that of the hospital/center. At the same time, the telemedicine setting allows for saving clinician activity time and patient waiting time and provides treatment in an ambulatory setting, avoiding going to the hospital and saving travel time.

In the Optimal pathway scenario time saving for HCPs are estimated at 0.21 FTE nurse staff per 100 patients/month with 1 therapy access, 0.06 FTE pharmacist and 0.02 FTE clinician.

### Costs

The results on monthly total costs for 100 patients with the probabilistic approach are summarized in Table 6*.* Per patient results can be consulted in S2 Table.

**Table 6:** Monthly total costs for 100 oncological patients in different pathways and in comparison with the current standard scenario.

Costs (€)	Standard	Drug change (Δ Standard)	Drug Day (Δ Standard)	Dedicated ambulatory (Δ Standard)	Optimal pathway (Δ Standard)
Healthcare Professional Active Time (IC), **Δ Standard**					
*Administrative* **Δ Standard**	€ 350 (265; 453) Ref	€ 350 (265; 453) **€ 0** **(0; 0)**	€ 332 (251; 430) **€ −17** **(−23; −13)**	€ 350 (265; 453) **€0** **(0; 0)**	€ 236 (179; 306) **€ −114** **(−147; −86)**
*Nurse* **Δ Standard**	€ 1,547 (1,149; 2,006) Ref	€ 1,057 (815; 1,310) **€ −490** **(−773; −264)**	€ 852 (657; 1,064) **€ −695** **(−1,000; −452)**	€ 877 (676; 1,095) **€ −670** **(−972; −431)**	€ 810 (624; 1,013) **€ −737** **(−1,048; −495)**
*Pharmacist* **Δ Standard**	€ 705 (528; 924) Ref	€ 349 (267; 445) **€ −357** **(−500; −246)**	€ 338 (259; 431) **€ −367** **(−512; −256)**	€ 349 (267; 445) **€ −357** **(−500; −246)**	€ 338 (259; 431) **€ −367** **(−512; −256)**
*Clinician* **Δ Standard**	€ 2,049 (1,563; 2,698) Ref	€ 2,049 (1,563; 2,698) **€ 0** **(0; 0)**	€ 2,049 (1,563; 2,698) **€ 0** **(0; 0)**	€ 2,049 (1,563; 2,698) **€ 0** **(0; 0)**	€ 1,897 (1,444; 2,499) **€ −151** **(−208; −106)**
*All* **Δ Standard**	€ 4,650 (3,989; 5,460) Ref	€ 3,804 (3,258; 4,481) **€ −846** **(−1,152; −586)**	€ 3,571 (3,042; 4,269) **€ −1,080** **(−1,397; −797)**	€ 3,624 (3,090; 4,320) **€ −1,027** **(−1,336; −745)**	€ 3,282 (2,785; 3,940) **€ −1,369** **(−1,699; −1,077)**
Infusion Chair Occupation Time (IC), **Δ Standard**	€ 986 (737; 1,265) Ref	€ 380 (282; 499) **€ −606** **(−804; −432)**	€ 380 (282; 499) **€ −606** **(−804; −432)**	€ 380 (282; 499) **€ −606** **(−804; −432)**	€ 380 (282; 499) **€ −606** **(−804; −432)**
Treatment (IC) **Δ Standard**	€ 280,583 (229,812; 334,402) Ref	€ 275,612 (239,071;318,655) **€ −4,971** **(−54,305; 47,689)**	€ 275,612 (239,071;318,655) **€ −4,971** **(−54,305; 47,689)**	€ 275,612 (239,071;318,655) **€ −4,971** **(−54,305; 47,689)**	€ 275,612 (239,071;318,655) **€ −4,971** **(−54,305; 47,689)**
Adverse event (IC) **Δ Standard**	€ 2,253 (1,787; 2,744) Ref	€ 800 (629; 1,006) **€ −1,453** **(−1,840; −1,099)**	€ 800 (629; 1,006) **€ −1,453** **(−1,840; −1,099)**	€ 800 (629; 1,006) **€ −1,453** **(−1,840; −1,099)**	€ 800 (629; 1,006) **€ −1,453** **(−1,840; −1,099)**
Total direct cost (IC) **Δ Standard**	€ 288,472 (237,836; 342,399) Ref	€ 280,596 (244,100; 324,299) **€ −7,876** **(−57,690; 44,437)**	€ 280,363 (243,861; 324,049) **€ −8,109** **(−57,922; 44,215)**	€ 280,416 (243,914; 324,098) **€ −8,056** **(−57,864; 44,264)**	€ 280,073 (243,564; 323,702) **€ −8.399** **(−58,270; 43,930)**
Patient Productivity Loss **Δ Standard**	€ 7,220 (5,244; 9,417) Ref	€ 5,300 (3,852; 6,885) **€ −1,920** **(−2,622; −1,324)**	€ 4,535 (3,304; 5,931) **€ −2,685** **(−3,552; −1,889)**	€ 4,664 (3,398; 6,085) **€ −2,557** **(−3,404; −1,792)**	€ 2,381 (1,764; 3,152) **€ −4,840** **(−6,352; −3,471)**
Caregiver Productivity Loss **Δ Standard**	€ 6,958 (4,659; 9,579) Ref	€ 5,108 (3,436; 6,956) **€ −1,851** **(−2,643; −1,202)**	€ 4,371 (2,917; 5,977) **€ −2,587** **(−3,613; −1,693)**	€ 4,495 (3,005; 6,146) **€ −2,463** **(−3,459; −1,606)**	€ 2,295 (1,526; 3,186) **€ −4,663** **(−6,394; −3,055)**
Total indirect cost (IC) **Δ Standard**	€ 14,179 (10,470; 18,229) Ref	€ 10,408 (7,691; 13,442) **€ −3,771** **(−5,067; −2,641)**	€ 8,906 (6,570; 11,520) **€ −5,273** **(−6,980; −3,794)**	€ 9,159 (6,745; 11,812) **€ −5,020** **(−6,671; −3,600)**	€ 4,676 (3,499; 6,087) **€ −9,503** **(−12,255; −7,014)**
Total cost **Δ Standard**	€ 302,651 (252,392; 354,653) Ref	€ 291,004 (254,193; 334,695) **€ −11,647** **(−60,892; 40,607)**	€ 289,269 (252,294; 332,694) **€ −13.382** **(−62,401; 38,958)**	€ 289.574 (252,646; 333,072) **€ −13,077** **(−62,102; 39,255)**	€ 284,750 (247,490; 328,377) **€ −17,901** **(−66,705; 34,780)**

Times are reported in hours.

^1^ Including travel time.

The cost analysis shows that the treatment cost item is lower when introducing SC formulation for 71% of patients: the cost decreases of €-17,901 per month per 100 patients (even if the result is not statistically relevant, due to the variability of the outcome, subject to change according to the dosage and the proportion of patients targeted for the two formulations). The direct cost related to management of adverse events decreases with the introduction of SC formulation, as the percentage of patients with PICC or PORT catheters decreased from 12% (standard scenario) to 4% (other scenarios) and from 67% to 25%, respectively. Looking at the total direct costs, it emerges how the implementation actions included in the Drug Change, Drug Day, Dedicated ambulatory, and Optimal pathways reduced both the cost of therapy and the cost of personnel and resources such as infusion chair occupancy time, leading to a total reduction in direct costs of, respectively: € −7,876, € −8,109, € −8,056 and € −8.399. This result also suffers from the lack of statistical significance due to the presence of the cost of treatment within it.

Looking at costs from a societal perspective, and thus including indirect costs related to lost productivity of patients and caregivers, all scenarios report cost savings compared to the standard: in the drug day the total savings correspond to €5,273 per month per 100 patients, in the dedicated ambulatory to €5,020 while in the Optimal pathway, the scenario that leads to the greatest savings, this comes to €9,503 per month per 100 patients.

Finally, the total cost (direct and indirect) is affected by treatment cost, and a non-statistically significant decrease of costs results in all the alternative scenarios versus the standard one: a saving of € 11,647 for the Drug Change scenario, a saving of €13.382 for the Drug Day, a saving of €13.077 for the Dedicated ambulatory, and a saving of € 17,901 for the Optimal pathway scenario.

### Sensitivity analysis

**Fig 2:**
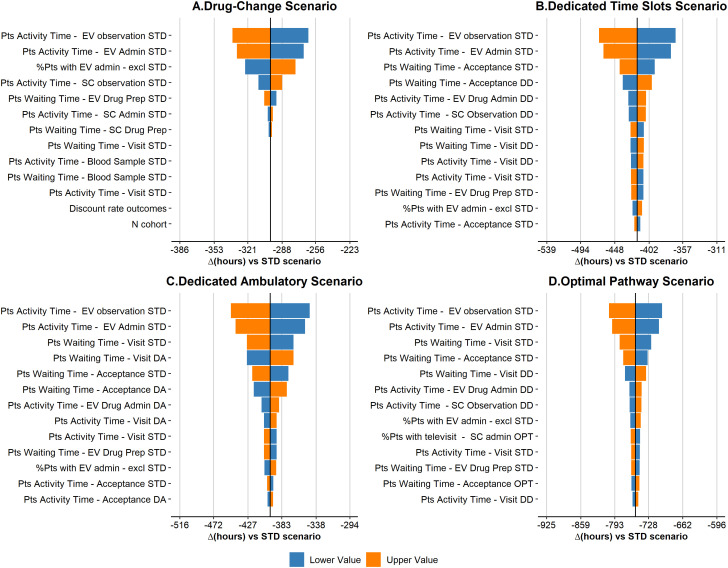
OWSA on monthly total patient time for every scenario on a standard number of 100 patients.

**Fig 3:**
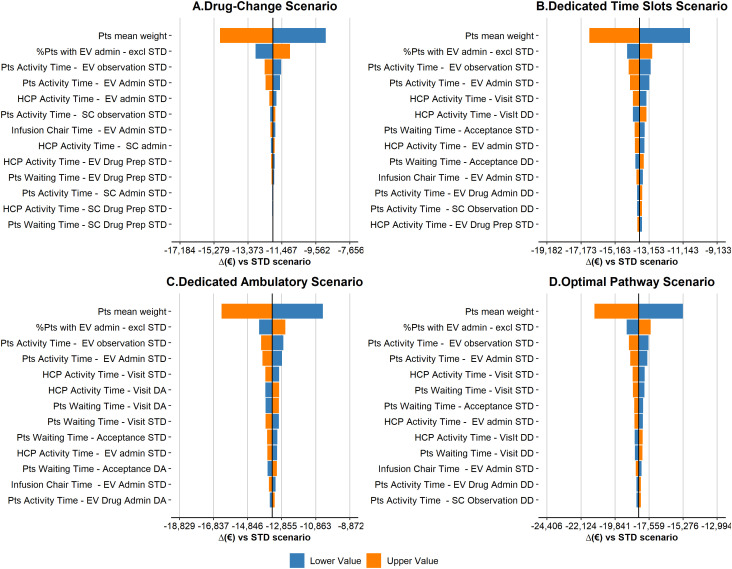
OWSA on monthly total costs for every scenario on a standard number of 100 patients.

The results of the sensitivity analysis on monthly total patient time and on monthly total costs per 100 patients are reported in Figs 2 and 3 respectively. The results of the sensitivity analysis on total HCP times and total direct costs are reported in S2 and S3 Figs.

Monthly total patient time was most affected by the patients’ activity time of the EV observation and administration in all the scenarios. A variation of ± 15% in the patient activity time in the EV observation phase would lead the difference from the standard scenario to range from − 263 to – 336 hours in the drug change scenario, from − 367 to − 470 in the drug day scenario, from − 347 to − 449 in the dedicated ambulatory scenario, and from – 702 to − 805 in the optimal pathway scenario. A variation of ± 15% in the patient activity time in the EV administration phase would lead the difference from the standard scenario to range from − 267 to – 331 hours in the drug change scenario, from − 373 to − 464 in the drug day scenario, from − 353 to −443 in the dedicated ambulatory scenario, and from − 708 to − 799 in the optimal pathway scenario. In the drug change scenario, the percentage of patients using EV formulation is one of the major parameters affecting patients’ time, while in the other alternative scenarios the impact is mitigated and improvement actions applied have a major impact.

Monthly total cost was most affected by patients’ mean weight and percentage of patients in EV therapy in all the scenarios. A variation of ± 15% of mean weight would lead the difference from the standard scenario to range from -€9008 to -€14943 in the drug change scenario, from -€10745 to -€16681 in the drug day scenario, from -€10438 to -€16737 in the dedicated ambulatory scenario, and from -€15287 to -€21223 in the optimal pathway scenario. While a variation of ± 15% of percentage of patients in EV therapy would lead the difference from the standard scenario to range from-€11009 to -€12942 in the drug change scenario, from -€12969 to -€14457 in the drug day scenario, from -€14170 to -€12642 in the dedicated ambulatory scenario, and from -€19059 to -€17451 in the optimal pathway scenario.

## Discussion

The analysis conducted through this model demonstrates how switching from the EV formulation to the SC formulation of the pertuzumab+trastuzumab fixed dose combination for subcutaneous administration (PH FDC SC) significantly improves the time and costs saved for patients undergoing therapy. The patients involved in this analysis are oncology patients who must undergo treatment administration every 3 weeks for 14 to 18 cycles depending on the setting (adjuvant therapy for early breast cancer or advanced/metastatic breast cancer, respectively). Their quality of life is thus significantly impacted by the pathology and treatment regimen [17] the reduction in throughput time and the consequent lower loss of productivity takes on even greater value.

The results on crossing times clearly show that the improvement actions introduced by the different alternative scenarios (including the SC formulation) save time and resources, both in terms of hospital staff and infusion chair occupancy. In the current Italian healthcare context, characterized by a shortage of healthcare personnel [18], especially doctors and nurses, these results assume important value for those managing hospital pathways. For example, time savings for nursing staff range from 20 hours per month in the drug change scenario (0.14 FTE) to about 30 in the optimal scenario (0.21 FTE), per 100 patients/month with one therapy access, while the infusion chair free time gained may be converted into new therapy infusions. Moreover, the introduction of SC formulation reduced infusion chair occupation time that may be converted into new therapy infusion when hospital staff is available. This reach of a significant recovery efficacy is one of the implementations that the Italian NHS needs most at a historical moment like the current one.

The saving in crossing or total times for hospital staff and patients and caregivers results in a reduction of associated direct and indirect costs. Also, the introduction of the SC formulation requires a lower cost of drug purchase that has a great impact on the total direct cost. Overall, the alternative scenarios demonstrated lower total costs compared with the Standard scenario, confirming the economic advantage and sustainability of the SC formulation.

Variability of model parameters have a great impact on the simulation results, so developed models can be adapted to specific oncological centers characteristics. Moreover, it includes alternative scenarios with different implementation scopes, such as the temporal dimension for the drug day scenario and the spatial dimension for the dedicated ambulatory scenario; this reflects the desire to provide hospital centers with more alternatives from which to choose and for which to opt, based on the center’s characteristics. Using our model, each hospital can simulate the results it would obtain by applying the four scenarios, evaluating which is feasible and most suitable based on the size and internal organization of the center, entering the center’s data as input, and simulating the different alternatives.

The optimal pathway scenario deserves further consideration. It has been conceived as an inspirational scenario, to be aimed at soon by following the directions that health policies are taking in Italy, such as the one encouraging telemedicine [19], and by taking advantage of new technologies for digitizing health processes, such as the one introduced in the acceptance phase of the scenario in question.

An extremely important element to be associated with the possibility of administering therapy subcutaneously is the fact that this mode of administration can also be carried out in an out-of-hospital setting, such as that of territorial health facilities, the implementation of which is one of the pivotal goals of current Italian health policies [13,19]. Among the various health care institutions that have outsourced this service, the city of Ferrara in the Emilia-Romagna Region has already introduced the administration of this therapy in territorial settings, with the dual advantage of getting geographically closer to patients without forcing them to travel to the hospital center, and of lightening the hospital’s workload, reserving for this setting only the most complex cases [20].

The study presents some limitations. The results reported in this study specifically refer to the treatment pathway for HER2 + breast cancer patients and do not encompass the broader spectrum of treatments, which may vary significantly across different oncology centers. To enhance the model’s applicability across facilities with diverse organizational structures and patient volumes, our findings were standardized to a cohort of 100 patients. This approach allows the results to be scaled and adapted to local clinical practice, accounting for the actual number of HER2 + patients treated and the specific allocation of HCPS’ time. Secondly, data are based on a study conducted on three centers with different dimensional levels and on direct experience of a panel of experts; this may limit the generalization of results. However, the developed model is a flexible tool that can be customized by individual facilities; thus, further real-world observational studies conducted within specific oncology centers would provide additional validation of the findings. Moreover, although the model includes the use of venous access devices (PICC and PORT) and related adverse events, based on real-world data, the prevalence of device use may vary across centers and could be higher in some clinical settings. Nevertheless, given the relatively limited economic weight of these components within the overall treatment pathway, their impact on the model results is expected to be modest. Lastly, while the study adopted both NHS and societal perspectives, some direct medical costs from the NHS perspective may not have been fully captured, such as waste disposal expenses, equipment maintenance or concomitant medications. Furthermore, the societal perspective was limited to indirect costs associated with patient and caregiver productivity loss. However, in a process-efficiency context, these estimates still provide a robust indication of the operational optimization achievable through different therapy administration pathways.

## Conclusions

This work demonstrates that transitioning from EV to SC administration for HER2+ breast cancer therapies, coupled with targeted pathway optimizations, yields significant time efficiencies and costs savings. The implementation of innovative measures, such as telemedicine, time slots and external blood sampling, underlines the transformative potential of integrating technology into patient management. Notably, the presented frameworks can help achieve substantial reductions in HCP workloads and patient waiting times, which is critical in addressing resource constraints in oncology centers.

By tailoring therapeutic pathways to the specific needs and capabilities of individual centers, this model provides a practical tool for decision-making in resource allocation and workflow optimization.

Future efforts should focus on validating these scenarios in real-world observational settings across heterogeneous oncology services, including both hospital and territorial contexts, to further confirm the magnitude of the observed organizational and economic benefits. As Italian healthcare policies increasingly emphasize telemedicine and decentralized care delivery, the findings of this study provide timely and actionable insights to guide policy implementation and improve the quality of care for HER2+ breast cancer patients.

## Supporting information

S1 Table Monthly crossing times (hours) for an oncological patient in different pathways and in comparison with the current standard scenario. (DOCX)

S2 Table Monthly total costs for an oncological patients in different pathways and in comparison with the current standard scenario. (DOCX)

S1 Fig The day-hospital administration pathway. The icons included in this figure were generated with assistance from the generative AI tool NotebookLM (Google LLC), while all text elements and the overall structure of the figure were created by the authors. The AI-generated icons were reviewed and edited by the authors before inclusion. According to Google Terms of Service, users retain rights over generated outputs. The authors take full responsibility for the accuracy, integrity, and final content of the figure.
(TIFF)

S2 Fig OWSA on monthly total HCP time for every scenario on a standard number of 100 patients. Pts – patients; HCP – healthcare professional; STD – standard scenario; DD – drug-day standard scenario; DA – dedicated ambulatory standard scenario; OPT – optimal pathway standard scenario; SC – subcutaneous; EV – endovenous; admin – administration; prep – preparation.
(TIFF)

S3 Fig OWSA on monthly total direct costs for every scenario on a standard number of 100 patients. Pts – patients; HCP – healthcare professional; STD – standard scenario; DD – drug-day standard scenario; DA – dedicated ambulatory standard scenario; OPT – optimal pathway standard scenario; SC – subcutaneous; EV – endovenous; admin – administration; prep – preparation.
(TIFF)
