# Supplementary material for: Decision tree model to assess consequences and costs associated with therapy administration pathways for patients with HER2+ breast cancer in Italian oncological centers
Source: PLoS One. 2026 Jul 24;21(7):e0351548. doi: 10.1371/journal.pone.0351548 (PMC13399340; doi:10.1371/journal.pone.0351548)
Supplement: S1 Table — (DOCX) [file pone.0351548.s001.docx]

**S1 Table. Monthly crossing times (hours) for an oncological patient in different pathways and in comparison with the current standard scenario**

| **Outcomes** | **Scheme** | | | | | **Difference (Δ Standard)** | | | |
| --- | --- | --- | --- | --- | --- | --- | --- | --- | --- |
|  | **Standard** | **Drug Change** | **Drug Day** | **Dedicated Ambulatory** | **Optimal Pathway** | **Drug Change** | **Drug Day** | **Dedicated Ambulatory** | **Optimal Pathway** |
| Healthcare Professional Active Time. hours |  |  |  |  |  |  |  |  |  |
| *Administrative* | 0.2  (0.1 ; 0.2) | 0.2  (0.1 ; 0.2) | 0.2  (0.1 ; 0.2) | 0.2  (0.1 ; 0.2) | 0.1  (0.1 ; 0.1) | 0.0  (0.0 ; 0.0) | 0.0  (0.0 ; 0.0) | 0.0  (0.0 ; 0.0) | -0.1  (-0.1 ; 0.0) |
| *Nurse* | 0.6  (0.5 ; 0.8) | 0.4  (0.4 ; 0.5) | 0.4  (0.3 ; 0.4) | 0.4  (0.3 ; 0.4) | 0.3  (0.3 ; 0.4) | -0.2  (-0.3 ; -0.1) | -0.3  (-0.4 ; -0.2) | -0.3  (-0.4 ; -0.2) | -0.3  (-0.4 ; -0.2) |
| *Pharmacist* | 0.2  (0.1 ; 0.2) | 0.1  (0.1 ; 0.1) | 0.1  (0.1 ; 0.1) | 0.1  (0.1 ; 0.1) | 0.1  (0.1 ; 0.1) | -0.1  (-0.1 ; -0.1) | -0.1  (-0.1 ; -0.1) | -0.1  (-0.1 ; -0.1) | -0.1  (-0.1 ; -0.1) |
| *Clinician* | 0.4  (0.3 ; 0.5) | 0.4  (0.3 ; 0.5) | 0.4  (0.3 ; 0.5) | 0.4  (0.3 ; 0.5) | 0.4  (0.3 ; 0.5) | 0.0  (0.0 ; 0.0) | 0.0  (0.0 ; 0.0) | 0.0  (0.0 ; 0.0) | 0.0  (0.0 ; 0.0) |
| *All* | 1.4  (1.3 ; 1.5) | 1.1  (1.0 ; 1.2) | 1.0  (0.9 ; 1.1) | 1.0  (0.9 ; 1.2) | 0.9  (0.8 ; 1.0) | -0.3  (-0.4 ; -0.2) | -0.4  (-0.5 ; -0.3) | -0.4  (-0.5 ; -0.3) | -0.5  (-0.6 ; -0.4) |
| Infusion Chair Occupation Time. hours | 2.4  (2.0 ; 2.9) | 0.9  (0.8 ; 1.1) | 0.9  (0.8 ; 1.1) | 0.9  (0.8 ; 1.1) | 0.9  (0.8 ; 1.1) | -1.5  (-1.9 ; -1.2) | -1.5  (-1.9 ; -1.2) | -1.5  (-1.9 ; -1.2) | -1.5  (-1.9 ; -1.2) |
| Patient in-hospital Time. hours |  |  |  |  |  |  |  |  |  |
| *Active Time* | 5.6  (5.0 ; 6.3) | 2.8  (2.5 ; 3.1) | 1.9  (1.7 ; 2.1) | 1.9  (1.7 ; 2.1) | 1.8  (1.6 ; 1.9) | -2.8  (-3.3 ; -2.3) | -3.7  (-4.3 ; -3.1) | -3.7  (-4.3 ; -3.1) | -3.9  (-4.5 ; -3.3) |
| *Waiting Time* | 3.3  (2.9 ; 3.7) | 3.1  (2.7 ; 3.5) | 2.8  (2.4 ; 3.1) | 3.0  (2.6 ; 3.4) | 1.7  (1.5 ; 2.0) | -0.2  (-0.3 ; -0.2) | -0.5  (-0.6 ; -0.4) | -0.3  (-0.4 ; -0.2) | -1.6  (-1.8 ; -1.3) |
| *Total Time* | 8.9  (8.1 ; 9.6) | 5.9  (5.4 ; 6.4) | 4.7  (4.3 ; 5.1) | 4.9  (4.5 ; 5.3) | 3.5  (3.1 ; 3.8) | -3.0  (-3.6 ; -2.5) | -4.2  (-4.8 ; -3.6) | -4.0  (-4.6 ; -3.4) | -5.4  (-6.1 ; -4.8) |
| Patient Total Time^1^. minutes | 11.2  (10.3 ; 12.1) | 8.3  (7.6 ; 9.0) | 7.1  (6.5 ; 7.7) | 7.3  (6.7 ; 7.9) | 3.7  (3.4 ; 4.1) | -3.0  (-3.6 ; -2.5) | -4.2  (-4.8 ; -3.6) | -4.0  (-4.6 ; -3.4) | -7.5  (-8.3 ; -6.7) |
| Caregiver Total Time^1^. minutes | 7.3  (5.7 ; 8.9) | 5.4  (4.2 ; 6.6) | 4.6  (3.6 ; 5.7) | 4.7  (3.7 ; 5.8) | 2.4  (1.9 ; 3.0) | -2.0  (-2.5 ; -1.4) | -2.7  (-3.4 ; -2.1) | -2.6  (-3.3 ; -2.0) | -4.9  (-6.1 ; -3.8) |

*Times are reported in hours.*

*1 Including travel time*
