# Supplementary material for: Decision tree model to assess consequences and costs associated with therapy administration pathways for patients with HER2+ breast cancer in Italian oncological centers
Source: PLoS One. 2026 Jul 24;21(7):e0351548. doi: 10.1371/journal.pone.0351548 (PMC13399340; doi:10.1371/journal.pone.0351548)
Supplement: S2 Table — (DOCX) [file pone.0351548.s002.docx]

**S2 Table. Monthly total costs for an oncological patients in different pathways and in comparison with the current standard scenario**

| **Costs (ϵ)** | **Scheme** | | | | | **Difference (Ref Standard)** | | | |
| --- | --- | --- | --- | --- | --- | --- | --- | --- | --- |
|  | **Standard** | **Drug Change** | **Drug Day** | **Dedicated Ambulatory** | **Optimal Pathway** | **Drug Change** | **Drug Day** | **Dedicated Ambulatory** | **Optimal Pathway** |
| Healthcare Professional Active Time |  |  |  |  |  |  |  |  |  |
| *Administrative* | € 4  (3 ; 5) | € 4  (3 ; 5) | € 3  (3 ; 4) | € 4  (3 ; 5) | € 2  (2 ; 3) | € 0  (0 ; 0) | € 0  (0 ; 0) | € 0  (0 ; 0) | € -1  (-2 ; -1) |
| *Nurse* | € 15  (12 ; 20) | € 11  (8 ; 13) | € 8  (7 ; 11) | € 9  (7 ; 11) | € 8  (6 ; 10) | € -5  (-7 ; -3) | € -7  (-10 ; -5) | € -7  (-9 ; -4) | € -7  (-10 ; -5) |
| *Pharmacist* | € 7  (5 ; 9) | € 3  (3 ; 4) | € 3  (3 ; 4) | € 3  (3 ; 4) | € 3  (3 ; 4) | € -4  (-5 ; -2) | € -4  (-5 ; -2) | € -4  (-5 ; -2) | € -4  (-5 ; -2) |
| *Clinician* | € 20  (15 ; 26) | € 20  (15 ; 26) | € 20  (15 ; 26) | 20.29  (15.13 ; 26.16) | € 19  (14 ; 24) | € 0  (0 ; 0) | € 0  (0 ; 0) | € 0  (0 ; 0) | € -1  (-2 ; -1) |
| *All* | € 46  (39 ; 54) | € 38  (32 ; 44) | € 35  (30 ; 42) | € 36  (30 ; 42) | € 33  (27 ; 38) | € -8  (-11 ; -6) | € -11  (-14 ; -8) | € -10  (-13 ; -7) | € -14  (-17 ; -11) |
| Infusion Chair Occupation Time | € 10  (7 ; 13) | € 4  (3 ; 5) | € 4  (3 ; 5) | € 4  (3 ; 5) | € 4  (3 ; 5) | € -6  (-8 ; -4) | € -6  (-8 ; -4) | € -6  (-8 ; -4) | € -6  (-8 ; -4) |
| Treatment | € 2.811  (2.352 ; 3.359) | € 2.762  (2.400 ; 3.162) | € 2.762  (2.400 ; 3.162) | € 2.762  (2.400 ; 3.162) | € 2.762  (2.400 ; 3.162) | € -49  (-534 ; 468) | € -49  (-534 ; 468) | € -49  (-534 ; 468) | € -49  (-534 ; 468) |
| **Total Direct Cost** | € 2.889  (2.427 ; 3.436) | € 2.812  (2.448 ; 3.215) | € 2.809  (2.446 ; 3.212) | € 2.810  (2.446 ; 3.213) | € 2.807  (2.443 ; 3.209) | € -78  (-564 ; 440) | € -80  (-566 ; 438) | € -79  (-566 ; 438) | € -83  (-569 ; 435) |
| Patient Productivity Loss | € 72  (54 ; 95) | € 53  (39 ; 69) | € 45  (34 ; 59) | € 47  (35 ; 61) | € 24  (17 ; 31) | € -19  (-26 ; -14) | € -27  (-36 ; -20) | € -26  (-34 ; -19) | € -49  (-64 ; -36) |
| Caregiver productivity loss | € 70  (48 ; 96) | € 51  (35 ; 71) | € 44  (30 ; 61) | € 45  (31 ; 63) | € 23  (16 ; 32) | € -19  (-27 ; -12) | € -26  (-37 ; -17) | € -25  (-35 ; -16) | € -47  (-64 ; -31) |
| **Total Indirect Cost** | € 142  (106 ; 185) | € 104  (78 ; 136) | € 89  (66 ; 116) | € 92  (68 ; 119) | € 47  (35 ; 61) | € -38  (-51 ; -27) | € -53  (-70 ; -38) | € -50  (-66 ; -36) | € -95  (-124 ; -70) |
| **Total Cost** | € 3,032  (2,570 ; 3,587) | € 2,916  (2,551 ; 3,323) | € 2,899  (2,536 ; 3,306) | € 2,902  (2,538 ; 3,309) | € 2,854  (2,490 ; 3,255) | € -116  (-602 ; 399) | € -133  (-615 ; 383) | € -130  (-612 ; 386) | € -178  (-656 ; 336) |
