# Supplementary figures and images for: Decision tree model to assess consequences and costs associated with therapy administration pathways for patients with HER2+ breast cancer in Italian oncological centers
Source: PLoS One. 2026 Jul 24;21(7):e0351548. doi: 10.1371/journal.pone.0351548 (PMC13399340; doi:10.1371/journal.pone.0351548)

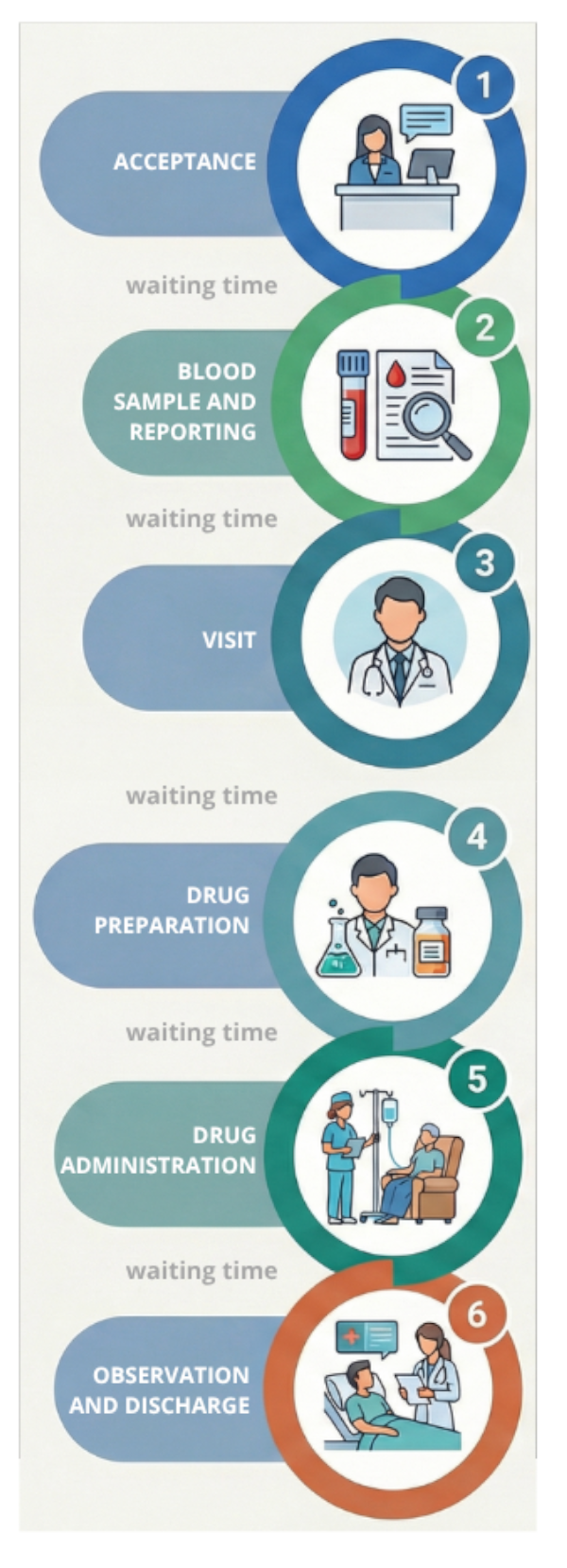

Supplement: S1 Fig — The icons included in this figure were generated with assistance from the generative AI tool NotebookLM (Google LLC), while all text elements and the overall structure of the figure were created by the authors. The AI-generated icons were reviewed and edited by the authors before inclusion. According to Google Terms of Service, users retain rights over generated outputs. The authors take full responsibility for the accuracy, integrity, and final content of the figure. (TIFF) [file pone.0351548.s003.tiff]

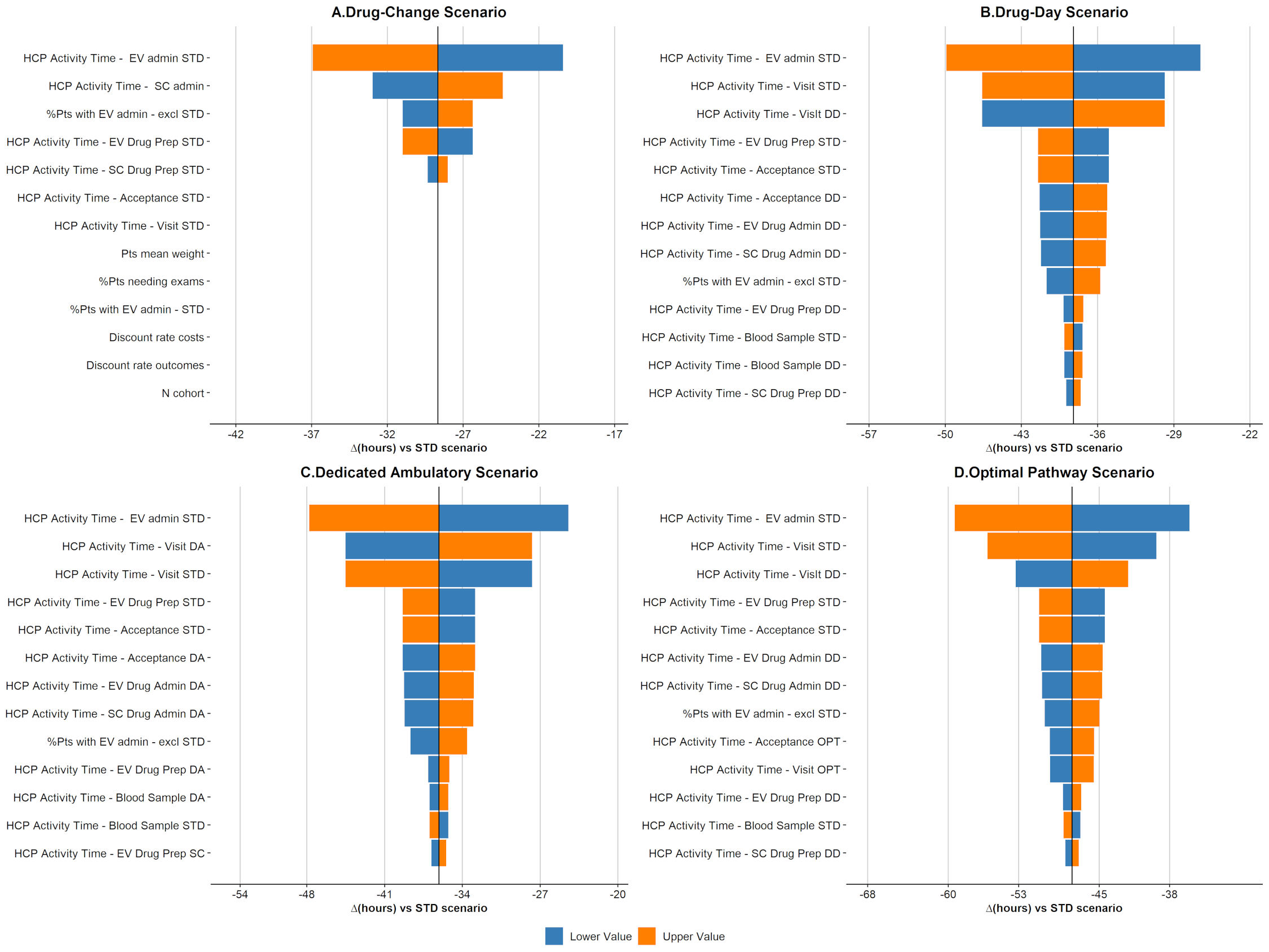

Supplement: S2 Fig — Pts – patients; HCP – healthcare professional; STD – standard scenario; DD – drug-day standard scenario; DA – dedicated ambulatory standard scenario; OPT – optimal pathway standard scenario; SC – subcutaneous; EV – endovenous; admin – administration; prep – preparation. (TIFF) [file pone.0351548.s004.tiff]

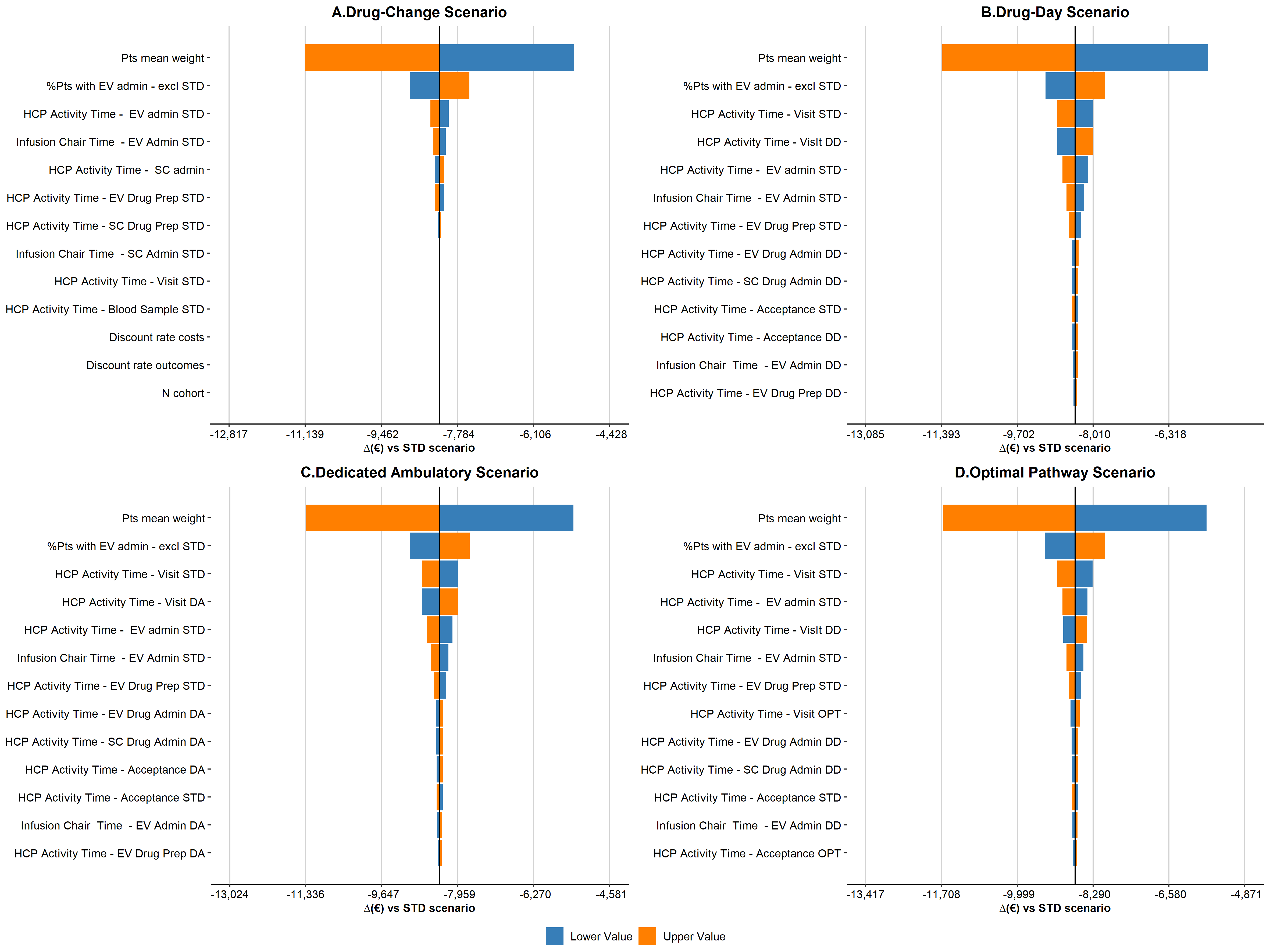

Supplement: S3 Fig — Pts – patients; HCP – healthcare professional; STD – standard scenario; DD – drug-day standard scenario; DA – dedicated ambulatory standard scenario; OPT – optimal pathway standard scenario; SC – subcutaneous; EV – endovenous; admin – administration; prep – preparation. (TIFF) [file pone.0351548.s005.tiff]
